# Delusions of Disseminated Fungosis

**DOI:** 10.1155/2014/458028

**Published:** 2014-12-25

**Authors:** Ian Gassiep, Paul Matthew Griffin

**Affiliations:** ^1^Department of Infectious Diseases, Mater Health Services and Mater Medical Research Institute, Brisbane, QLD 4101, Australia; ^2^School of Medicine, University of Queensland, Brisbane, QLD 4101, Australia; ^3^The QIMR Berghofer Medical Research Institute, Brisbane, QLD 4101, Australia

## Abstract

*Introduction*. Delusional infestation is a rare monosymptomatic hypochondriacal psychosis according to The Diagnostic and Statistical Manual of Mental Disorders (5th ed.; DSM-5; American Psychiatric Association, 2013). It can be a primary disorder or associated with an underlying psychological or physical disorder. It commonly presents as delusional parasitosis, and less than 1% may be fungi related. We present this case as it is a rare presentation of a rare condition. *Case Presentation*. Our patient is a 60-year-old Caucasian man who presented with a 7-year history of delusional infestation manifested as a disseminated fungal infection. He had previously been reviewed by multiple physicians for the same with no systemic illness diagnosed. After multiple reviews and thorough investigation we diagnosed him with a likely delusional disorder. As is common with this patient cohort he refused psychiatric review or antipsychotic medication. *Conclusion*. A delusion of a disseminated fungal infestation is a rare condition. It is exceedingly difficult to treat as these patients often refuse to believe the investigation results and diagnosis. Furthermore, they either refuse or are noncompliant with treatment. Multidisciplinary outpatient evaluation may be the best way to allay patient fears and improve treatment compliance.

## 1. Introduction

The delusion of infestation (DI) is a primary psychiatric disorder, a patient's fixed belief that he or she is infested with an animate or inanimate object causing distressing symptomatology, often pruritus [1–5]. Documented delusions of parasitosis date as far back as 1894 [1]. Since then there have been numerous revisions in terminology including Ekbom's syndrome [2], delusions of parasitosis [3], Morgellons syndrome [4], and most recently delusional disorder of the somatic delusional type, a form of monosymptomatic hypochondriacal psychosis [5]. This disorder is relatively rare and it is estimated that for every seven years of practice a dermatologist will see one case [6]. Given the need for patient-compliant treatment there has been a call for revised nomenclature given its pejorative nature, to that of pseudoparasitic dysesthesia [7]. It would appear that the broader term of delusions of infestation is more appropriate for our patient, as we report the rare case involving delusions of a disseminated fungal infection. There are very few case reports of fungal infestation, and only one previously cited German dissertation estimated delusional infestation due to fungi at approximately 1% [8–10]. To our knowledge, this is the first reported case of a persistent delusional disorder involving multiorgan fungosis and fungemia.

## 2. Case

A 54-year-old man was referred to our infectious diseases outpatients clinic by his primary care physician for ongoing management of a possible disseminated fungal infection.

**Figure 1:**
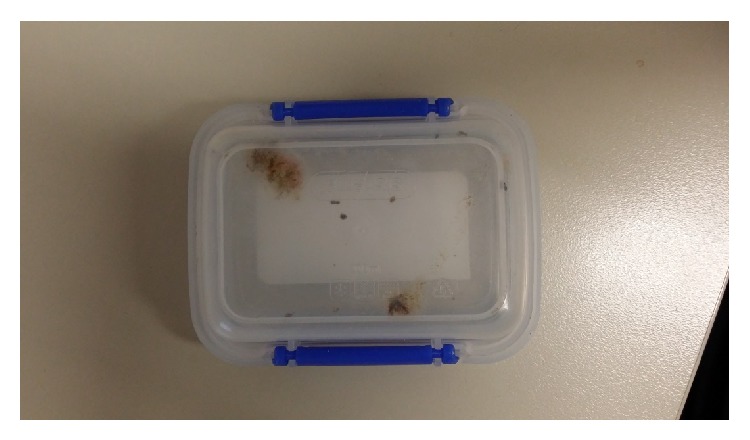
Specimen containing “proof” of the patient's infestation.

The patient's presenting complaint was a 7-year history of a pervasive “fungus” infection throughout his body. He states this infection moves from one part of his body to the next at random. He described the intermittent sensation of webbing over his eyes and further described formication that travels up from his feet to his scalp. He also stated that when he showered in the mornings and sunlight came into his bathroom, he could see the fungal spores and felt he was reinfecting his lungs and eyes. During the consultation he repeatedly attempted to rub the fungus off his skin to prove its existence.  Figure 1 shows material the patient collected from his fingernails after vigorous scratching, which he incubated in water at room temperature for 6 weeks.

Initially the patient was seen after a glancing injury to the eye for which he was diagnosed and treated for fungal keratitis to good effect by an ophthalmologist. Since this event he states the infection has spread and had been unsuccessfully treated. His primary care physician had reviewed him on numerous occasions for the same complaint and treated him with both topical and oral antifungal preparations including Clotrimazole and Griseofulvin. He had furthermore attempted oral antibiotic therapy with cephalexin and metronidazole, all with no effect. Following failed attempts at treatment the patient states he was reviewed by three dermatologists and two infectious disease physicians with no cause for his symptoms found. Unsatisfied with his consultations, he attended a naturopath whom he states performed confirmatory blood tests diagnosing a fungemia. Unfortunately he was unable to produce evidence of this visit or results of investigations performed. The patient's past medical history is notable for untreated Hepatitis C genotype 2b infection, a past Hepatitis B infection, and a distant history of intravenous drug use for which he did not require opioid therapy. He takes no oral medications and applies tea-tree oil to his skin when required, as he felt this assisted in controlling his fungal infection.

Over the course of three reviews there were no abnormal examination findings of this patient. For completeness of consultation investigations including white cell count, C-reactive protein, human immunodeficiency virus, T-lymphocyte counts, cryptococcal antigen, galactomannan, and a skin scraping for microscopy and culture were performed, results of which were all within normal range or negative. On presentation of these results to the patient he was thankful the tests had been performed, but unfortunately he was incredulous as to the negative results. Once rapport had been developed we delved further into the patient's recent psychiatric history. During his second consultation he admitted to a depressed mood, decreased appetite, increased sleep, episodes of unprovoked teariness, hopelessness, and previous episodes of self-harm. He did however deny any active thoughts of self-harm or suicide. When the subject of depression was broached the patient wholeheartedly agreed but felt his depression stemmed from inadequate treatment of his fungal infection. At this point he agreed to a psychiatric review as well as a course of an antidepressant; however on review several months later he has failed to attend his appointments or commence his medication.

## 3. Discussion

Delusional infestation can be stratified into two groups. Primary DI is a pure delusional disorder, whereas secondary DI is associated with another psychiatric disorder most commonly schizophrenia, dementia, and depression and can also be associated with diabetes mellitus and organic brain pathology such as neoplasm, infection, or stroke [11, 12]. Our patient clearly meets criteria for a depressive disorder, and as such he may be classified as suffering from a form of secondary DI [5]. However, in this instance it is difficult to ascertain which one occurred first.

As is typical with patients manifesting delusions of infestation the firm belief is not easily altered. Our patient's review by multiple nonpsychiatric clinicians is a typical feature of his underlying diagnosis [13–15]. His refusal to see a psychiatrist or commence antipsychotic medication is another typical feature [16, 17]. The need for our patient to attempt to show us “proof” of infestation by vigorously rubbing his skin can be seen as a variant of the pathognomonic “matchbox” sign [11, 15, 16]. This sign is usually manifest by patients bringing in a specimen as proof of infestation. The patient does have a past history of illicit drug use as well as hepatitis B and C infection. Various medications, illicit drugs, and medical conditions have been associated with delusional infestation [8].

Our patient's delusion was reinforced by his naturopath. We are uncertain as to what tests may have been performed in the patient and do not know how the diagnosis of a fungemia was made. There is very little literature regarding the use of alternative medicine with regard to delusional infestation. It would stand to reason that this patient population seeking multiple clinical reviews would also seek opinions from nonmedical therapists, especially if they are able to support the delusion. Unfortunately this can be significantly detrimental to the patient especially in accepting the true diagnosis and treatment options.

With regard to treatment, atypical antipsychotics such as risperidone or olanzapine are recommended as first-line agents [17]. Pimozide, an antipsychotic with good clinical efficacy, is no longer used due to its adverse effects profile which includes Parkinsonism, prolonged QT interval, and drug-drug interactions [12]. A major difficulty is patient acceptance of the psychological component to the disorder. With this in mind commencement of antipsychotic medication is challenging. Techniques such as explaining that there is a possible chemical imbalance related to previous infection and that the antipsychotic medication is not specifically for treatment of schizophrenia in this instance may improve patient receptiveness to therapy [18]. Our patient has been hesitant to therapy but was at least contemplative to the idea once these techniques were employed. Aside from pharmacotherapy, psychotherapy and ongoing multidisciplinary treatment will likely be of greatest benefit. There is an emerging field of dermatology dealing specifically with psychocutaneous diseases, namely psychodermatology. The main practice aim is to combine dermatology and psychiatry or psychology, in an outpatient clinical setting. This combined clinic aids in allaying patient fear regarding psychiatric review and aims to improve patient-doctor communication and compliance with treatment and may well be useful for long term management of these patients [19].
